# Croatian National Data and Comparison with European Practice: Data from the Cardiac Resynchronization Therapy Survey II Multicenter Registry

**DOI:** 10.1155/2018/3479846

**Published:** 2018-10-25

**Authors:** Sandro Brusich, Ivan Zeljković, Nikola Pavlovic, Ante Anić, Zrinka Jurišić, David Židan, Marina Klasan, Zlatko Čubranić, Kenneth Dickstein, Cecilia Linde, Camilla Normand, Sime Manola

**Affiliations:** ^1^Rijeka University Hospital Centre, Rijeka, Croatia; ^2^Sestre Milosrdnice University Hospital Centre, Zagreb, Croatia; ^3^County Hospital Zadar, Zadar, Croatia; ^4^Split University Hospital Centre, Split, Croatia; ^5^Stavanger Univeristy Hospital, Stavanger, Norway; ^6^Karolinska University Hospital, Stockholm, Sweden

## Abstract

**Aims:**

The Cardiac Resynchronization Therapy (CRT) Survey II was conducted between October 2015 and December 2016 and included data from 11088 CRT implantations from 42 countries. The survey's aim was to report on current European CRT practice. The aim of this study was to compare the Croatian national CRT practice with the European data.

**Methods:**

Five centres from Croatia recruited consecutive patients, in a 15-month period, who underwent CRT implantation, primary or an upgrade. Data were collected prospectively by using online database.

**Results:**

A total of 115 patients were included in Croatia, which is 33.2% of all CRT implants in Croatia during the study period (total *n*=346). Median age of the study population was 67 (61–73) years, and 21.2% were women. Primary heart failure (HF) aetiology was nonischemic in 61.1% of patients, and HF with wide QRS was the most common indication for the implantation (73.5%). 80% of patients had complete left bundle branch block, and over two-third had QRS ≥150 ms. Device-related adverse events were recorded in 4.3% of patients. When compared with European countries, Croatian patients were significantly younger (67 vs. 70 years, *p*=0.012), had similar rate of comorbidities with the exception of higher prevalence of hypertension. Croatian patients significantly more often received CRT-pacemaker when compared with European population (58.3 vs. 29.9%, OR 3.27, 95%CI 2.25–4.74, *p* < 0.001).

**Conclusion:**

Our data indicate strict selection of patients among HF population and adherence to guidelines with exception of higher proportion of CRT-pacemaker implantation. This is likely to be influenced by healthcare organization and reimbursement issues in Croatia.

## 1. Introduction

The advantages of cardiac resynchronization therapy (CRT) on long-term clinical treatment of symptomatic heart failure (HF) patients (NYHA II-IV) with reduced left ventricular ejection fraction (LVEF) and ventricular dyssynchrony have been constantly confirmed [1–3]. Surveys and registries include all eligible consecutive patients, providing very useful “real-world” clinical data [1, 2]. These data complement the results from randomized clinical trials (RCT), in which high-risk patients are usually not included [2–5]. The first European Cardiac Resynchronization Therapy (CRT) Survey was conducted in 2008, as a joint project of the Heart Failure Association (HFA) and the European Heart Rhythm Association (EHRA) of the European Society of Cardiology (ESC) [6]. It was a 6-month snapshot survey which included more than 2400 patients with CRT implantation from 13 ESC member countries. It gave valuable insights into CRT clinical practice and emphasized underutilization of resynchronization therapy at that time, also showing that large numbers of CRTs were implanted not applying the guidelines recommendations [6]. In 2013 and 2016, ESC issued new guidelines on cardiac pacing and CRT as well as on acute and chronic HF management [7, 8]. European CRT Survey II was designed and implemented on the basis of first CRT Survey, but did not include any follow-up [9]. It included 42 ESC member countries with the aim to gather demographic and clinical data on the HF patient selection, CRT implantation, and follow-up practice. This survey provided information relevant for assessing healthcare resource utilization, the impact of new guidelines on daily clinical practice, and adherence to CRT guidelines [10]. The aim of this study is to report Croatian national data on resynchronization therapy practice and compare it with the rest of the European data gathered in the same multicentre CRT II Survey.

## 2. Materials and Methods

The CRT II Survey was designed and conducted as a joint project of the EHRA and HFA, and its design and rationale have been published previously [9]. Between October 2015 and December 2016, all consecutive patients that underwent a primary CRT implantation or an upgrade to a CRT-system were eligible for inclusion, regardless of the success of the procedure. Data were collected prospectively by using online database. A central database was created and maintained at the data management centre (IFH, Heidelberg University, Germany), which also performed the data analyses. The overall results of the CRT Survey II were recently published [10]. Five Croatian centres participated in the survey (out of 8 implanting CRT devices): University Hospital Centre *Rijeka*, University Hospital Centre *Sestre milosrdnice*, University Hospital Centre *Zagreb*, University Hospital Centre *Split*, and County Hospital *Zadar*.

The European CRT Survey II included two internet-based questionnaires [8, 9]. The first one was the questionnaire completed by a recruiting centre and included description of hospital type, reference area size, invasive procedures and device implantations details, cardiac facilities, types of imaging equipment employed, the number and speciality of implanting physicians, and the follow-up options provided, and ultimately the type and source of hospital reimbursement. The second questionnaire was an electronic case report form (eCRF) for each patient included in the survey. It was initiated prior to implantation, and included patient's demographic and clinical data, as well as procedural and short-term postprocedural details.

Ethics approval from the relevant Ethics Committee in Croatia was obtained. This study was conducted according to the Declaration of Helsinki.

### 2.1. Statistical Analysis

Continuous variables were presented as median with interquartile range or means with standard deviations. Continuous variables were compared with nonparametric Mann–Whitney *U* test. Categorical variables were presented as absolute values and/or percentages and were compared using Pearson's *χ*^2^ test. Descriptive statistics were calculated for the available cases. Two-sided *p* value of < 0.05 was considered significant. Statistical analysis was carried out using SASã statistical software, version 9.1 (Cary, North Carolina, USA).

## 3. Results

During the 15-month enrolment period (October 2015–December 2016), a total of 115 patients (1.03%) were recruited in Croatia, out of 11088 recruited by 288 centres in 42 ESC member countries participating in the CRT II Survey. This was 33.2% of all CRT implantations in Croatia during this period (total number = 346) [11, 12].

### 3.1. Preprocedural Data

The median age of Croatian CRT population was 67 (IQR 61–73) years, and 21.2% were women. Mean body mass index (BMI) was 27.0 ± 5.3 kg/m^2^, and 22.1% of patients had normal body weight (BMI 18–25 kg/m^2^). 75.2% of patients had hypertension, almost one-third had diabetes mellitus, and 26.5% had chronic kidney disease. 46% of patients were hospitalized due to heart failure (HF) during the past year before the implantation. No patient was asymptomatic or classified as NYHA functional class I, whereas 48.2% were in class II, 50% in class III, and 1.8% in NYHA class IV (Table 1). Primary HF aetiology was nonischemic in 61.1% of included patients. Concerning baseline heart rhythm and ECG, 23% of patients were in atrial fibrillation at the time of implantation, and 15% had AV II/III degree block. 80% of patients had complete left bundle branch block (LBBB), over two-third had QRS ≥150 ms, and 8.3% had QRS <120 ms. Heart failure with wide QRS complex was the most common indication for CRT implantation by far (73.5% of cases) followed by LV dysfunction with an indication for an implantable cardioverter-defibrillator (ICD) (27.4%) (Table 1).

The mean NT-pro BNP value was 3447.5 ± 3270.8 pg/mL; however, it was assessed in only 15.6% of patients. According to echocardiography, mean LVEDD was 65.6 ± 8.5 mm, mean was LVEF 29.1 ± 7.3% with 25.6% of patients having LVEF > 35%, and no patient had LVEF > 50%. 87.1% of patients had some degree of mitral regurgitation, mostly moderate (50%).

### 3.2. Procedural Data

The CRT-P device was implanted in 58.3% and CRT-D in 41.7% of patients. The procedure was done by an electrophysiologist in 88.7% of cases, and mostly performed in the catheterization laboratory (55.7%). The median duration of the procedure was 100 min (IQR 80–120) with a median fluoroscopy time of 15 min (IQR 10–22). 92.2% of patients received prophylactic antibiotics preprocedurally. Positioning of the leads was reported by the operator. The right ventricular (RV) lead was almost always placed before the left ventricular (LV) lead (98.3% of cases) and was most often positioned in the RV apex (75.4%). The LV lead was placed in the lateral position in 92.2%, and in the posterior position in 7% of patients. Operators mostly used the bipolar LV lead (95.7%) and the multipolar lead in 4.3% of cases. Regarding the procedural complications, no patient died during the implantation. There were no bleeding complications, 5 patients had coronary sinus dissections (4.3%), and 1 patient had pneumothorax (0.9%). Detailed procedural data are given in Table 2.

### 3.3. Postprocedural Data

The mean length of hospital stay was 6.8 ± 7.5 days. 1 patient died during hospitalization following the implantation due to noncardiovascular reasons. Device-related adverse events after the implantation were recorded in 4.3% of patients. Lead displacement was observed in 2.7%, dominantly on behalf of the LV lead (66.7%) (Table 3). Phrenic nerve stimulation was observed in 2 patients, but it did not require a new procedure. There was no infection related to the CRT implantation during the hospitalization. The mean paced QRS duration after optimization was 113 ± 19 ms. Detailed data on medication therapy at discharge are given in Table 3. Follow-up was planned for every patient only at the implanting centre.

### 3.4. Comparison with European Practice

When compared with other European countries (total of 10973 patients), Croatian CRT population (*n*=115) was significantly younger (67 vs. 70 years, *p*=0.012), had lower proportion of patients > 75 years (22.1 vs. 32.1%, OR 0.6, 95%CI 0.38–0.94, *p* < 0.001), and had similar low implantation rate in women (21.2 vs. 24.3%, *p*=0.445). Baseline and preprocedural data of both groups are given in Table 1. Regarding comorbidities, Croatian patients had higher prevalence of arterial hypertension (OR 1.73, 95%CI 1.12–2.65, *p*=0.012) and lower prevalence of previous coronary revascularization (OR 0.56, 95%CI 0.36–0.86, *p*=0.006) and previously implanted devices (OR 0.39, 95%CI 0.23–0.68, *p* < 0.001). Regarding the NYHA functional class, there was no significant difference overall. HF with wide QRS as the main indication for CRT implantation was significantly more often in Croatian patients (OR 1.86, 95%CI 1.22–2.83, *p*=0.003). Croatian patients had similar rate of LVEF <35%, complete LBBB, and QRS < 150 ms in comparison with average European population. According to echocardiography, mean LVEF was similar between the groups, even when divided in groups: on <35%, 35–50%, and >50%. However, Croatian patients had higher LV end-diastolic diameter (65.6 vs. 63.5 mm, *p*=0.039) and higher prevalence of moderate mitral regurgitation (OR 2.81, 95%CI 1.90–4.16, *p* < 0.001).

The comparison of procedural data between the groups is given in Table 2. Croatian patients were significantly more likely to receive CRT-P device when compared with European average (58.3 vs. 29.9%, OR 3.27, 95%CI 2.25–4.74, *p* < 0.001). There were significantly less upgrade procedures to CRT system (13.3 vs. 28%, OR 0.39 (0.23–0.68, *p* < 0.001). In Croatia, the implantation procedure was more often elective (OR 1.72, 95%CI 1.02–2.88, *p*=0.037), done more often by an electrophysiologist (OR 2.36, 95%CI 1.32–4.21, *p*=0.014) and almost 4 times more likely to be done in a catheterization laboratory (OR 3.78, 95%CI 2.61–5.47, *p* < 0.001). There was no difference regarding both the total duration of the procedure and fluoroscopy time between the groups. In Croatia, the multipolar LV lead was used significantly less often (OR 0.03, 95%CI 0.01–0.08, *p* < 0.001), but during coronary sinus venogram, the usage of balloon occlusion was almost 2-fold more common (OR 1.87, 95%CI 1.28–2.73, *p*=0.001). Defibrillation threshold testing was carried out less often (0.9 vs. 4.8%, OR 0.17, 95%CI 0.02–1.25, *p*=0.049). Regarding procedural complications, there were no significant differences between the groups (*p*=0.847). Total length of hospital stay was longer in Croatia (6.8 vs. 6.3 days, *p*=0.002). Postprocedurally, the duration of QRS after optimization was significantly lower in Croatian patients (113 vs. 138 ms, *p* < 0.001). There were no differences regarding the incidence of major adverse events connected to implantation during the hospitalization. There was a statistically significant difference between the groups regarding the discharge medication therapy and the follow-up planning. The complete postprocedural data are given in Table 3.

## 4. Discussion

**Figure 1:**
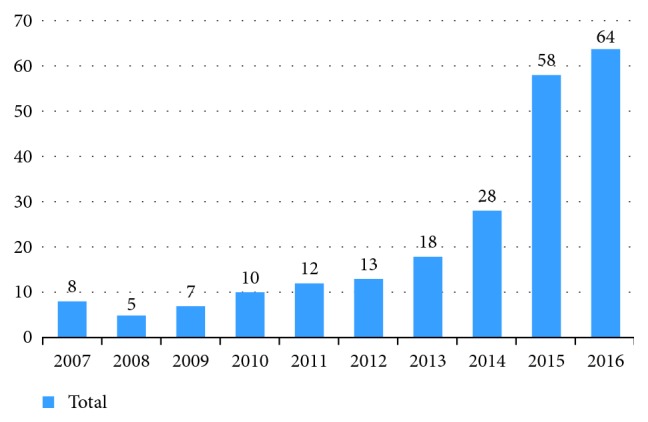
Number of CRT implantations per million inhabitants in Croatia in the last decade.

European CRT Survey II was a 15-month snapshot survey, carried out by EHRA and HFA, and it provides a robust overview of the current clinical practice and guideline adherence regarding CRT across a wide range of centres in 42 ESC member countries [9, 10]. Croatia is a small country with around 4.5 million inhabitants and a gross domestic product (GDP) of 11'858 USD, and it allocates 7.8% of its GDP for healthcare [12]. In the last decade, a significant increase in CRT implantation number has been recorded. For comparison, in 2008 when CRT Survey I was conducted, only 5 CRTs per million inhabitants were implanted in Croatia, while in 2016, that number grew to 64, which is an increase of more than 900%, but still low when compared with Western European countries (Figure 1) [11, 12]. 5 out of 8 centres that implant CRT participated in the CRT II Survey. They are all classified as medium or high volume centres, and they did 88.5% of all CRTs implantation in 2016 [11]. However, only 33.2% of all implanted cases in the study period were reported, which could have biased the national results [11, 13]. This is also true for other countries participating in the survey. Therefore, comparison between Croatia and Europe could be accurate; however, Croatian results could have been slightly different if all patients from all implanting centres were included.

The data show that Croatian patients were significantly younger when compared with the average European population, however with rather high prevalence of comorbidities, especially of hypertension which is in line with previous epidemiological studies of general population in Croatia [14]. A consistent finding was the low proportion of women receiving CRTs both in Croatia and across Europe, even though women have better survival after CRT implantation [1, 6, 8, 10, 15]. Croatian patients had more elective hospitalization for CRT implantation (85%), although almost half of the patients (46%) were hospitalized due to HF during the past year before the implantation. This is probably due to organizational arrangements concerning hospitals' budget limitations and reimbursement issues and a need to screen the population with the CRT indication [10, 12, 16, 17]. Also, patients in Croatia received more CRT-pacemaker (CRT-P) devices compared with other European countries (58 vs. 30%) despite a significant proportion having an indication for a CRT-defibrillator according to the current guidelines [7]. We can speculate that this is mainly due to healthcare organization, reimbursement, and budget restrictions [17–19]. However, this is in compliance with the new cohort studies and meta-analysis on sudden cardiac death (SCD) in HF population, which stress the importance of pharmacological therapy and CRT-P in SCD prevention [20–22]. Even stricter adherence to guidelines is notable when the patient selection is closely studied: majority have nonischemic aetiology of HF, 80% complete LBBB, two-third QRS >150 ms, and less than 25% were > 75 years old. All aforementioned are the significant predictors of good CRT response and better long-term clinical outcome [1–3, 6–8, 20–22]. This kind of selection from HF population with a CRT indication is presumably caused by limited budget. Likewise, mostly bipolar electrodes were implanted, and more often in comparison with other European countries, even though quadripolar leads are more cost-effective in the long term [23].

In Croatia, although procedures did not last significantly longer than the European average, most patients had the LV lead positioned laterally, which is the golden standard, but not easily achievable [1–3, 24, 25]. Moreover, this could explain the very narrow postprocedural QRS in Croatian patients, significantly narrower in comparison with the European counterparts, which is a predictor of clinical outcome, but could also explain higher incidence of coronary sinus dissection [2, 3, 22–25]. Procedures were mostly done in the catheterization laboratory, probably influenced by organizational characteristics of implanting centres. However, since these centres do most of CRT implantation in Croatia, it was expected for them to be provided in a specialized electrophysiology implanting room. Also, most of the procedures were done by electrophysiologist, which is expected due to complexity of the procedure and the total number of CRTs implanted through the year [11–13]. Over the years, the number of all cardiac devices implanted by surgeons has decreased significantly in Croatia [11, 13].

The reported perioperative complication rate was low, which is in line with the fact that centres that participated are medium or high volume centres [12, 26, 27]. The Croatian group had no bleeding complications, which are the most common, and which is in line with the lower rate of patients on antiplatelet and/or anticoagulant therapy [7, 26–28]. Regarding the discharge medication therapy, Croatian patients were significantly more often prescribed with Warfarin as anticoagulation therapy, since novel anticoagulants (NOAC) are only partially covered by the public health insurance [18, 19, 29].

The Croatian CRT population when compared with the average European population was significantly younger and had similar rate of comorbidities and even higher rate of hypertension. More than 30% of all CRT implants during the study period were included in this survey, which makes the data highly representative for Croatia. Most of the patients that received CRT had one or more significant predictors of good clinical outcome: complete LBBB, HF with wide QRS as a main indication, QRS > 150 ms, nonischemic aetiology of cardiomyopathy, and younger than 75 years. Like in the overall survey, many but not all CRT implantations were made in accordance with Guidelines indication IA [10]. The overall Croatian implantation practice showed high adherence to current guidelines which was similar to European practice, which is not the case in the CRT Survey I [2, 10]. Also, the CRT-pacemaker system was implanted in significantly higher proportion. There are probably several reasons for that. First, budget limitations significantly limit the number of more expensive CRT-D devices, which partially explains this difference [17–19]. Second, such patients benefit the most from CRT which modifies the HF condition and have less added benefit from adding ICD to CRT [20, 21]. This selectiveness of physicians when choosing patients for CRT implantation among HF population is also probably driven by budget limitations [17–19, 29].

In conclusion, Croatian national data from the European CRT Survey II provides clinicians and healthcare providers with data useful for improving HF patient management and could drive our efforts for better reimbursement policy with the goal of providing CRT therapy to a higher number of patients.

## Figures and Tables

**Table 1:** Baseline characteristics and preprocedural data of Croatian CRT population and comparison with the European population.

	Croatia	Europe	*p* value	OR (95% CI)
*Demographics*				
Age	66.1 ± 11.0	68.6 ± 10.8	0.017	
Men	78.8 (89/113)	75.7 (8277/10939)	0.445	
BMI	28.1 ± 4.2	27.9 ± 5.0	0.162	

*History*				
Hypertension	75.2 (85/113)	63.8 (6877/10787)	0.012	1.73 (1.12–2.65)
Diabetes mellitus	30.1 (34/113)	31.4 (3394/10808)	0.765	
Obstructive lung disease	8.8 (10/113)	12.1 (1305/10809)	0.295	
Atrial fibrillation	33.6 (38/113)	40.9 (4421/10807)	0.117	
Paroxysmal	28.9 (11/38)	34.8 (1537/4421)	0.344	
Persistent	21.1 (8/38)	22.3 (986/4421)		
Permanent	50.0 (19/38)	42.3 (1870/4421)		
Chronic kidney disease (<60)	26.5 (30/113)	31.2 (3365/10794)	0.291	
Prior revascularization (CABG or PCI)	50.4 (57/113)	75.3 (8145/10813)	0.006	0.56 (0.36–0.86)
HF hospitalisation during last year	46.0 (52/113)	46.5 (5026/10804)	0.915	
Prior device (PPM, ICD)	13.3 (15/113)	28.0 (3044/10879)	<0.001	0.39 (0.23–0.68)
Primary HF aetiology			0.477	
Ischaemic	38.1 (43/113)	44.6 (4832/10840)		
Nonischaemic	61.1 (69/113)	49.7 (5384/10840)		
Others	0.9 (1/113)	5.8 (624/10840)		

*ECG*				
Heart rate (beats/min)	76 ± 19	72 ± 16	0.028	
Sinus rhythm	76.1 (86/113)	69.1 (7410/10723)	0.071	
Atrial fibrillation	23.0 (26/113)	25.7 (2752/10723)		
PR interval (ms)	182 ± 47	189 ± 50	0.337	
AV block II or III	15.0 (17/113)	19.0 (2009/10587)	0.289	
QRS duration (ms)	154 ± 24	157 ± 27	0.436	
QRS duration <120 ms	8.3 (9/108)	7.4 (702/9427)		
QRS duration 120–130 ms	5.6 (6/108)	5.3 (499/9427)		
QRS duration 130–150 ms	18.5 (20/108)	18.7 (1759/9427)		
QRS duration 150–180 ms	48.1 (52/108)	47.0 (4434/9427)		
QRS duration >180 ms	19.4 (21/108)	21.6 (2033/9427)		

*QRS morphology*				
LBBB	80.0 (88/110)	75.2 (7750/10307)	0.245	
RBBB	8.2 (9/110)	6.6 (679/10307)	0.503	
Others	11.8 (13/110)	18.2 (1878/10307)	0.083	

*CRT indication*				
Heart failure with wide QRS	73.5 (83/113)	59.8 (6467/10810)	0.003	1.86 (1.22–2.83)
HF or LV dysfunction and an indication for ICD	27.4 (31/113)	48.1 (5197/10810)	0.001	0.41 (0.27–0.62)
PM indication + expected pacing dependency	21.2 (24/113)	22.8 (2470/10810)	0.685	
Evidence of medical dyssynchrony	4.4 (5/113)	11.6 (1255/10810)	0.018	0.35 (0.14–0.87)
Clinical evaluation			0.158	
NYHA I	0.0 (0/112)	3.4 (370/10736)		
NYHA II	48.2 (54/112)	37.5 (4029/10736)		
NYHA III	50.0 (56/112)	54.5 (5853/10736)		
NYHA IV	1.8 (2/112)	4.5 (484/10736)		

*Echocardiography*				
Mean LV ejection fraction (%)	29.1 ± 7.3	28.4 ± 8.2	0.314	
<35%	74.3 (84/113)	76.6 (8198/10692)		
35–50%	25.6 (29/113)	21.5 (2299/10692)		
>50%	0.0 (0/113)	1.8 (195/10692)		
LV end-diastolic diameter (mm)	65.6 ± 8.5	63.5 ± 9.1	0.039	
Mitral regurgitation			<0.001	
Mild	39.2 (40/102)	46.5 (4604/9898)		0.74 (0.50–1.11)
Moderate	50.0 (51/102)	26.2 (2595/9898)		2.81 (1.90–4.16)
Severe	7.8 (8/102)	6.9 (682/9898)		1.15 (0.56–2.38)
None	2.9 (3/102)	20.4 (2017/9898)		0.12 (0.04–0.37)

*Laboratory results*				
NT-pro BNP (pg/mL)	3447.5 ± 3270.8^*∗*^	5111.8 ± 8144.1	0.725	
Hemoglobin (g/L)	13.6 ± 1.6	13.3 ± 1.8	0.079	
Creatinine (μmol/L)	107.1 ± 36.7	114.1 ± 65.9	0.417	

Values are % (*n*) for categorical and mean ± standard deviation or median (25^th^–75^th^ percentile) for continuous variables. BMI: body mass index; CABG: coronary artery bypass grafting; PCI: percutaneous coronary intervention; HF: heart failure; PPM: permanent pacemaker; ICD: implantable cardioverter-defibrillator; LBBB: left bundle branch block; RBBB: right bundle branch block; LV: left ventricle; BNP: brain-type natriuretic peptide. ^∗^NT-pro BNP was measured only in 18 patients in Croatia (15.6% of patients).

**Table 2:** Procedural data of Croatian CRT population and comparison with the European practice.

	Croatia	Europe	*p* value	OR (95% CI)
Elective procedure	85.1 (97/114)	76.9 (8325/10832)	0.038	1.72 (1.02–2.88)
Location of procedure			<0.001	
Cath lab	55.7 (64/115)	24.9 (2654/10643)		3.78 (2.61–5.47)
Dedicated EP lab	20.0 (23/115)	30.8 (3277/10643)		0.56 (0.36–0.89)
Device implantation lab	22.6 (26/115)	33.6 (3575/10643)		0.58 (0.37–0.90)
Operating theatre	1.7 (2/115)	10.2 (1082/10643)		0.16 (0.04–0.63)
Operator			0.014	
Electrophysiologist	88.7 (102/115)	76.9 (8200/10664)		2.36 (1.32–4.21)
Heart failure physician	0.0 (0/115)	5.1 (541/10664)		/
Invasive cardiologist	1.7 (2/115)	12.5 (1328/10664)		0.12 (0.03–0.50)
Surgeon	0.9 (1/115)	4.3 (463/10664)		0.19 (0.03–1.39)
Others	8.7 (10/115)	1.2 (132/10664)		7.6 (3.88–14.86)
Duration of procedure (min)	100 (80, 120)	90 (65, 120)	0.163	
Fluoroscopy time (min)	15 (10, 22)	14 (8, 22)	0.11	
RV lead position			0.002	
Apex	75.4 (86/114)	61.1 (6194/10139)		1.96 (1.27–3.00)
Septum	23.7 (27/114)	36.6 (3706/10139)		0.54 (0.35–0.83)
RVOT	0.9 (1/114)	2.4 (239/10139)		0.37 (0.05–2.64)
LV lead position			0.7	
Anterior	0.9 (1/115)	4.4 (446/10185)		0.19 (0.03–1.37)
Lateral	92.2 (106/115)	84.0 (8559/10185)		2.24 (1.13–4.43)
Posterior	7.0 (8/115)	11.6 (1180/10185)		0.57 (0.28–1.17)
LV lead type			<0.001	
Unipolar	0.0 (0/115)	0.7 (77/10486)		/
Bipolar	95.7 (110/115)	41.7 (4368/10486)		30.81 (12.5–75.5)
Multipolar	4.3 (5/115)	57.6 (6041/10486)		0.03 (0.01–0.08)
Coronary venogram performed	99.1 (114/115)	91.4 (9522/10414)	0.003	10.68 (1.49–76.5)
Venogram with occlusion	62.3 (71/114)	46.9 (4415/9408)	0.001	1.87 (1.28–2.73)
Test shock	0.9 (1/115)	4.8 (505/10531)	0.049	0.17 (0.02–1.25)
Periprocedural complications	5.2 (6/115)	5.6 (618/10973)	0.848	
Bleeding	0.0 (0/115)	1.0 (108/10973)	0.772	
Pocket haematoma	0.0 (0/115)	0.8 (85/10973)	0.285	
Pneumothorax	0.9 (1/115)	1.0 (111/10973)	0.879	
Pericardial tamponade	0.0 (0/115)	0.3 (28/10973)	0.587	
Coronary sinus dissection	4.3 (5/115)	1.9 (209/10973)	0.058	
Type of the device (%)			<0.001	
CRT-P	58.3 (67/115)	29.9 (3189/10654)		3.27 (2.25–4.74)
CRT-D	41.7 (48/115)	70.1 (7465/10654)		0.31 (0.21–0.44)
Prophylactic antibiotics	92.2 (106/115)	98.7 (10421/10557)	<0.001	0.15 (0.08–0.31)

Values are % (*n*) for categorical and mean ± standard deviation or median (25^th^–75^th^ percentile) for continuous variables. EP: electrophysiology; RV: right ventricle; RVOT: RV outflow tract; LV: left ventricle; CRT-P: cardiac resynchronization therapy pacemaker system; CRT-D: cardiac resynchronization therapy cardioverter-defibrillator system.

**Table 3:** Postprocedural data of Croatian CRT population and comparison with the European average.

	Croatia	Europe	*p* value	OR (95% CI)
Hospital mortality	0.9 (1/111)	0.4 (44/10734)	0.409	

*Device-related complications*				
Lead displacement	2.7 (3/110)	1.7 (185/10720)	0.424	
RV	0.0 (0/3)	31.6 (55/174)	0.241	
LV	66.7 (2/3)	52.3 (91/174)	0.621	
Atrial	33.3 (1/3)	19.0 (33/174)	0.531	
Lead malfunction	0.0 (0/110)	0.2 (23/10720)	0.627	
Phrenic nerve stimulation	1.8 (2/110)	1.1 (121/10720)	0.497	
Infection	0.0 (0/110)	0.6 (60/10706)	0.431	
Stroke	0.0 (0/110)	0.1 (6/10706)	0.804	
Worsening of HF	0.0 (0/110)	0.7 (78/10706)	0.369	
Arrhythmias	0.9 (1/110)	1.2 (127/10706)	0.789	
Total length of hospital stay	6.8 ± 7.5	6.3 ± 11.4	0.002	
Mean-paced QRS duration (ms)	113 ± 19	138 ± 24	<0.001	

*Medical therapy at discharge*				
Diuretic	89.1 (98/110)	81.0 (8523/10525)	0.031	1.92 (1.05–3.50)
ACE inhibitor/ARB	84.1 (90/107)	86.4 (9073/10496)	0.484	
Aldosterone antagonist	71.8 (79/110)	63.1 (6603/10463)	0.059	
Beta blocker	94.5 (104/110)	88.9 (9368/10538)	0.06	
Digoxin	5.6 (6/107)	10.5 (1094/10437)	0.101	
Calcium channel blocker	6.7 (7/105)	9.0 (939/10426)	0.404	
Amiodarone	35.5 (38/107)	17.1 (1787/10440)	<0.001	2.67 (1.79–3.98)
Ivabradine	0.0 (0/108)	5.7 (593/10435)	0.011	/
Other antiarrhythmic agents	1.9 (2/108)	1.7 (179/10423)	0.256	
Oral anticoagulant	46.4 (51/110)	46.6 (4877/10467)	0.962	
Vitamin K antagonist	86.3 (44/51)	70.1 (3419/4877)	0.012	2.68 (1.20–5.96)
Dabigatran	3.9 (2/51)	6.7 (325/4877)	0.434	
Rivaroxaban	3.9 (2/51)	12.5 (609/4877)	0.065	
Apixaban	5.9 (3/51)	10.4 (506/4877)	0.294	
Edoxaban	0.0 (0/51)	0.4 (18/4877)	0.664	
Platelet inhibitor	25.2 (29/115)	43.9 (4817/10973)	<0.001	0.43 (0.28–0.66)
ASA	24.8 (27/109)	41.5 (4330/10438)	<0.001	0.46 (0.30–0.72)
Clopidogrel	6.4 (7/109)	12.4 (1297/10438)	0.058	0.48 (0.22–1.04)
Ticagrelor	0.9 (1/109)	1.3 (135/10438)	0.729	0.71 (0.10–5.10)
Dual antiplatelet therapy	5.5 (6/109)	9.3 (975/10438)	0.17	
OAC plus P2Y12 inhibitor	1.8 (2/110)	4.2 (438/10510)	0.219	
Triple therapy	1.8 (2/110)	2.1 (216/10511)	0.862	
Device follow-up planned	95.7 (110/115)	97.6 (10708/10973)	0.181	0.54 (0.22–1.35)
At implanting centre	100.0 (110/110)	86.2 (9235/10708)	<0.001	

Values are % (*n*) for categorical and mean ± standard deviation or median (25^th^–75^th^ percentile) for continuous variables. RV: right ventricle; LV: left ventricle; ACE: angiotensin-converting enzyme; ARB: angiotensin-receptor blocker; ASA: acetylsalicylic acid; OAC: oral anticoagulation.

## Data Availability

The summarized data used to support the findings of this study have been deposited in the international registry. Also, the summarized data used to support the findings of this study are included within the article. The individual data used to support the findings of this study are restricted by the Croatian laws in order to protect patients' privacy. Data are available from Ivan Zeljkovic, corresponding author, for researchers who meet the criteria for access to confidential data.
